# Cervical-Vaginal Microbiome and Associated Cytokine Profiles in a Prospective Study of HPV 16 Acquisition, Persistence, and Clearance

**DOI:** 10.3389/fcimb.2020.569022

**Published:** 2020-09-25

**Authors:** Anna-Barbara Moscicki, Baochen Shi, Hazel Huang, Emma Barnard, Huiying Li

**Affiliations:** ^1^Division of Adolescent and Young Adult Medicine, Department of Pediatrics, University of California, Los Angeles, Los Angeles, CA, United States; ^2^Department of Molecular and Medical Pharmacology, David Geffen School of Medicine, Crump Institute for Molecular Imaging, University of California, Los Angeles, Los Angeles, CA, United States

**Keywords:** microbiome, inflammation, cytokines, cervix, human papillomavirus

## Abstract

Persistent human papillomavirus (HPV) infections is necessary for the development of cervical cancers. Consequently, understanding the biologic mechanisms resulting in clearance is key in cancer prevention. Similar to other mucosal sites, it is expected that the local microbiome plays a significant role in shaping the immune response responsible for HPV clearance. Using cervical wash repository samples from a prospective study of HPV in women, this study investigates the microbiome and its associated inflammatory milieu during HPV 16 pre-acquisition, persistence and clearance states. For comparison, samples from women with no history of HPV ever during the study period were selected. We showed that 9 of 13 inflammatory cytokines were found to be significantly increased in the immediate post-clearance visit compared to the pre-acquisition or infection visits. *Gardnerella vaginalis* was associated with higher levels of inflammatory cytokines. Women with no history of HPV infection had similar cytokine profiles as those with HPV 16 post-clearance. This *in vivo* study documented an immune response shortly after HPV 16 clearance. *G. vaginalis* appeared to be involved in shaping this immune response. The appearance of *G. vaginalis* may have resulted from a shift from anti-microbial to anti-viral immune response with loss of bacterial control. The similar high levels of cytokines seen in women with no history of HPV suggest that a certain level of inflammatory surveillance is required to maintain an HPV negative state. This data may inform therapies such as probiotics or pro-inflammatory agents for treatment of persistent HPV.

## Introduction

The oncogenic capabilities of high risk human papillomavirus (hrHPV), the cause of cervical cancers, are well-known and include cell cycle deregulation, interference with telomerase activity and apoptosis (Doorbar, 2006; Wise-Draper and Wells, 2008). Yet, studies have shown that the vast majority of HPV infections are cleared and do not lead to cervical cancer (CC) (Moscicki et al., 1998; Kjaer et al., 2010). This is true even for HPV 16, the most common type associated with cervical and other anogenital cancers. *In vitro* and *in vivo* studies suggest that clearance of HPV infections is directed by a host anti-viral immune response (Scott et al., 1999; Woodworth, 2002; Doorbar, 2006; Farhat et al., 2009; Daud et al., 2011; Stanley, 2012). There is emerging evidence that the vaginal microbiome may be involved in modulating the local host immune response, as seen in other mucosal sites (Doerflinger et al., 2014; Anahtar et al., 2015). This influence can be either protective or harmful (Doerflinger et al., 2014; Mitchell and Marrazzo, 2014; Mittal et al., 2014; Zevin et al., 2016). Compared to the gut microbiome, the vaginal microbiome is considered less complex. Increased diversity of the vaginal microbiome that is *Lactobacillus*-non-dominated has been associated with diseased states while lower diversity with microbiome that is dominated by *Lactobacillus* species is associated with vaginal health (Doerflinger et al., 2014). Vaginal microbiome dysbiosis has been associated with pro-inflammatory states that result in the influx of immune infiltrates which in turn can release potential carcinogens (Doerflinger et al., 2014; Mittal et al., 2014; Anahtar et al., 2015). Consequently, the balance between a good and bad inflammatory response is critical to understand.

Two recent meta-analysis and systematic reviews that examined predominantly cross-sectional studies found that vaginal dysbiosis defined by studies using microscopy or molecular techniques were associated with prevalent cervical HPV and squamous intra-epithelial lesions (SIL) (Brusselaers et al., 2019). More specifically, vaginal microbiome (VM) dominated by non-*Lactobacilli* species or *L. iners* were more likely to have hrHPV or dysplasia/cancer than VM dominated by *L. crispatus* (Norenhag et al., 2020). In general, most studies have inconsistent findings when examining specific bacterial species (Gao et al., 2013; Brotman et al., 2014; Mitra et al., 2015; Shannon et al., 2017). This is not too surprising since none of these bacteria exist in a microbiome vacuum.

Since HPV clearance is protective against cervical cancer, delineating the microbiome and immune environment before acquisition, during persistence and after clearance can potentially provide insight into mechanisms associated with viral control that could be translated into therapies or diagnostic markers. We had the unique opportunity to examine biorepository samples prospectively collected from women participating in a longitudinal study of the natural history of HPV that characterized women for HPV DNA status at 4–6 month intervals for up to 25 years. This design allowed us to select samples from women with a documented incident HPV 16 infection with eventual clearance defined by the disappearance of viral detection. We focused on HPV 16 since this is the most common HPV type associated with cervical cancers. We had also collected metadata of the women at each visit. Our rich clinical data allowed us to adjust for potential confounders, such as antibiotic use and co-infections. We investigated longitudinal changes in the cervical-vaginal microbiome and associated cytokine profiles with HPV 16 pre-acquisition, during persistence, and after clearance.

## Materials and Methods

### Study Population

Repository samples were obtained from the San Francisco HPV cohort study of which the study design and population has been detailed previously (Moscicki et al., 2001, 2010, 2014). Briefly, healthy women of 13–21 years of age with normal cytology were asked to participate in a natural history study of HPV, which ran from 1990 to 2014. Women were seen at 4–6 month intervals and detailed history on sexual and substance use behaviors, contraceptive use, antibiotic use, intravaginal medications or products and menstrual history was obtained at each visit. Testing for *Chlamydia trachomatis* and *Neisseria. gonorrhea* was performed annually or if symptomatic. Wet mounts and pH were obtained at each visit for diagnosis of *Trichomonas vaginalis*, yeast and bacterial vaginosis. In addition, cervical washes (CW) with 5 ml of normal saline were obtained at each visit. CW were immediately placed onto ice and frozen at −20° C within 4 h until transported on ice to the lab weekly. Two ml were removed for HPV DNA testing and the remaining 3 ml were divided into 3 cryovials for −80° C storage. HPV testing was performed based on PCR amplification using Roche Linear Array and an in-house Luminex platform (Moscicki et al., 1998, 2001; Farhat et al., 2015). The primary study was approved by the Committee on Human Research, University of California, San Francisco and patients consented to participate and to place their samples in repository. This study used samples from the repository and was approved by the Institutional Review Board of the University of California, Los Angeles.

We chose 14 women who had acquired HPV 16, showed persistence for at least 8 months (three consecutive visits) and then cleared with no subsequent positive tests for HPV 16 (e.g., serial HPV results − − −+++−). The age range at first sampling ranged from 16 to 25 years, the number of visits with eligible samples ranged from 5 to 8 (see below) and the majority were white or Latina (see Supplementary Table 1). We selected the following visits for each woman: three HPV16 negative visits prior to HPV16 acquisition including the immediate visit prior to acquisition, 3 visits during persistence, and 2 visits following clearance (Supplementary Table 2). Of the potential 112 samples from the 14 subjects, 14 were excluded because the patient was pregnant or had a sexually transmitted infection or yeast vaginitis or had used antibiotics or intravaginal medications or products currently or anytime during the interval between visits. Since it was impossible to select samples from the same time during the menstrual cycle for all the women, we selected several visits prior to acquisition in an attempt to account for the women's normal menstrual cycle variability that has been previously described (Gajer et al., 2012). The average interval between the last pre-acquisition visit and first positive was 8.7 months, between the first and last positive visits was 15.2 months, and between the last positive and first post-clearance visit was 8.9 months.

Since women who acquire HPV 16 may be uniquely different, we identified 8 women who never acquired any HPV during their observation time in the study (~20% of our larger cohort of 1,500 women) (Supplementary Table 1). Visits with exclusion criteria listed above were not used. Four visits were chosen with similar time spans to the 14 women with HPV 16 infection (Supplementary Table 2).

In order to examine the integrity of the older samples, we first chose 10 samples from different women with a diagnoses of bacterial vaginosis (BV) by Nugent Score; (Nugent et al., 1991) 5 were 10 years or older stored at −70° C and 5 were more recent (within last 4 years). We compared the microbiome to that of published data and found that the microbiome make-up of the older and new samples were similar to that of the published data (Human Microbiome Project Consortium, 2012).

### Microbiome Sample Preparation, Sequencing, and Analysis

Genomic DNA was extracted from 250 μl CW aliquot using PowerSoil DNA Isolation Kit (MO BIO Laboratories, Carlsbad, CA, USA) combined with bead beating. The hypervariable regions V1-V3 of the 16S ribosomal RNA (rRNA) gene were amplified using universal primers 27F and 534R according to the protocol developed by the Human Microbiome Project (Human Microbiome Project Consortium, 2012). Nextera indices were used for multiplexing. 16S rRNA amplicon libraries were purified, quantified and pooled for sequencing on Illumina MiSeq platform. Paired-end reads of 300 bp were obtained for each sample. A negative control was included in each set of the PCR reactions. A data cleaning process was applied to all sequence data prior to analysis, and microbial taxonomic composition was determined as described in Shi et al. (2016).

The microbial community diversity (alpha diversity) was measured by Shannon index, and was calculated using QIIME (Caporaso et al., 2010). The samples were clustered and assigned to the vaginal community state types based on the abundance profiles of the bacterial taxa using hierarchical clustering in Cluster 3.0 (de Hoon et al., 2004). The samples were assigned based on the microbiome composition as defined in previous studies (Ravel et al., 2011; Brotman et al., 2012) (Supplementary Figure 1). Three states were identified: microbiome dominated by *L. crispatus* (DLC), microbiome dominated by *L. iners* (DLI), and those not dominated by any *Lactobacillus* species (NDL) (Ravel et al., 2011).

### Multiplex Assays for Chemokines and Cytokines

A second 250 μl CW aliquot was used for the multiplex assay of chemokines and cytokines. Inflammatory markers were chosen based on our finding from previous publications (Scott et al., 2013) and their potential role in HPV clearance including anti-viral, regulatory and pro-inflammatory properties (Stanley, 2010). After centrifugation, 25 μl of the cell-free supernatant was used for quantifying interferon factor interferon (IFN)-α2, IFN-γ, IL-1α, IL-1β, IL-4, IL-5, IL-6, IL-8, IL-10, IL-12(p70), IL-13, macrophage inflammatory protein (MIP)-1α, and tumor necrosis factor (TNF)-α. Specimens were tested in duplicate using MilliPlex MAP Human Cytokine/Chemokine immunoassay kits (Millipore Corporation, Billerica, MA) according to the manufacturer's instructions (Hwang et al., 2015). Plates were run on LuminexMagPix (Luminex, Austin, TX). Regression curves (5-parameterlogistic) were fit to determine concentrations in pg/mL using MiraiBio MasterPlex QT version 2.5 analysis software (Hitachi Solutions America, Ltd.).

### Statistical Analyses

Wilcoxon rank-sum test with two-tailed distribution was used in testing differences in the CW microbiome composition, the microbial diversity, as well as the expression levels of cytokines between the immediate (1st) post-clearance and each of the 3 pre-acquisition and 3 infection visits and 2nd post-clearance visit from women who acquired HPV 16. For women with no history of HPV infection, all the samples as a control group were compared to the samples in the immediate (1st) post-clearance from women who acquired HPV 16. The dynamics of the CW microbiome were analyzed based on the probability of transitions among the 3 states (DLC, DLI, NDL) using the Markov chain modeling (DiGiulio et al., 2015). The associations between the vaginal microorganisms and cytokines were calculated using Pearson correlation coefficients. We used the median values of the relative abundances of bacterial species and the cytokine expression levels among the samples at each visit in the calculation of the correlations between bacterial species and cytokine expression over the clinic visits.

## Results

### Microbiome Composition and Dynamics

Study population, definitions for acquisition, persistence and clearance, and selection of samples from the 14 women who had an incident of HPV 16 infection and clearance used for this study are described in Methods and in Supplementary Tables 1, 2.

**Figure 1:**
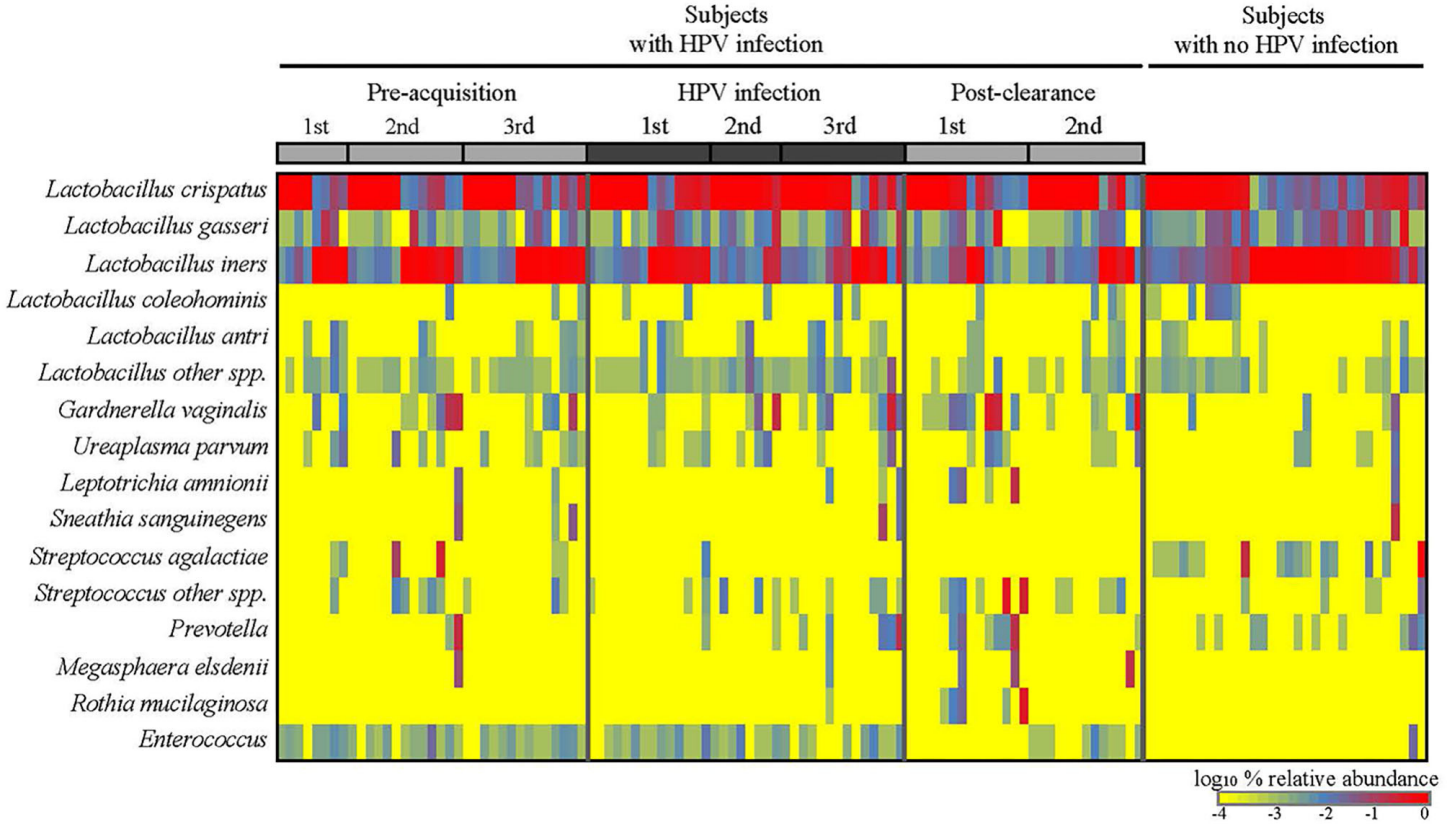
Cervical-vaginal microbiome composition of the women across different HPV states. Relative abundances of the dominant bacterial species detected in the samples by HPV states are as follows: three pre-acquisition visits, two or three visits during infection, one immediate (1st) post-clearance visit, and one later (2nd) post-clearance visit. Samples from the women with no history of HPV infection ever were analyzed as a comparison.

**Figure 2:**
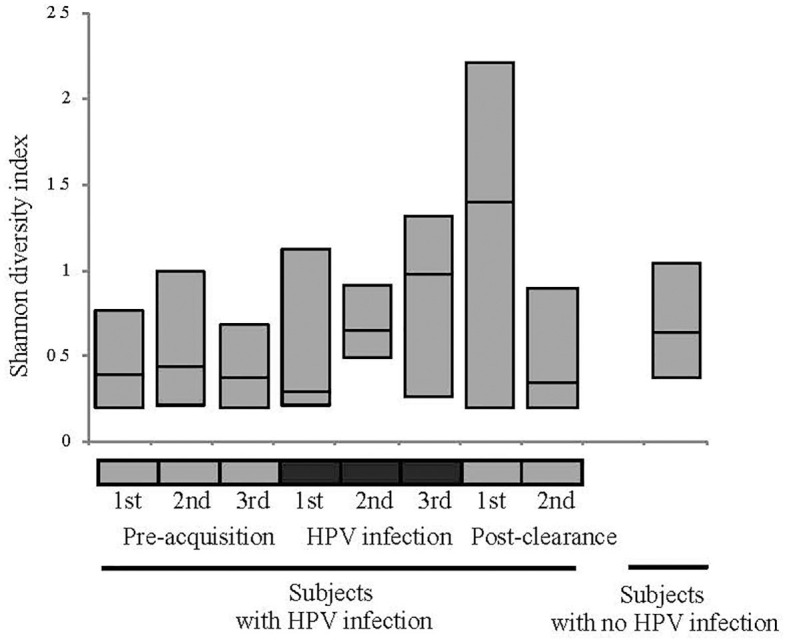
Alpha diversity measured by Shannon index of the microbiome in different HPV states. See Figure 1 definition for HPV states. No statistical differences in the alpha diversity were observed between HPV states or between HPV states and women with no history of HPV.

We first analyzed the microbiome composition of all samples grouped based on the HPV 16 status (Figure 1 and Supplementary Figure 1). The relative abundance of the corresponding 3 CW microbiome community states [dominated by *L. crispatus* (DLC), dominated by *L. iners* (DLI), and non-dominated *Lactobacillus* (NDL)] are shown in Supplementary Figure 2. We found that, compared to all the pre-acquisition visits combined, more samples from the immediate post-clearance visits had NLD microbiota, a state associated with a higher relative abundance of *Gardnerella vaginalis*, and fewer samples had DLI (Fisher exact test; *p* = 0.003) (Supplementary Figure 3). The same trend was also observed when samples from post-clearance were compared to those from the women with no history of HPV (WNHPV); more samples from the immediate post-clearance had NLD microbiota compared to WNHPV (Fisher exact test; *p* = 0.02), indicating that the microbiome at post-clearance visits was at dysbiosis. Alpha diversity measured by Shannon index, which is an indicator of the evenness, and richness of the microbial community, found no statistical differences between the two groups with different HPV 16 status (Figure 2), however, the post-clearance visit showed a trend of increased diversity compared to the other states.

**Figure 3:**
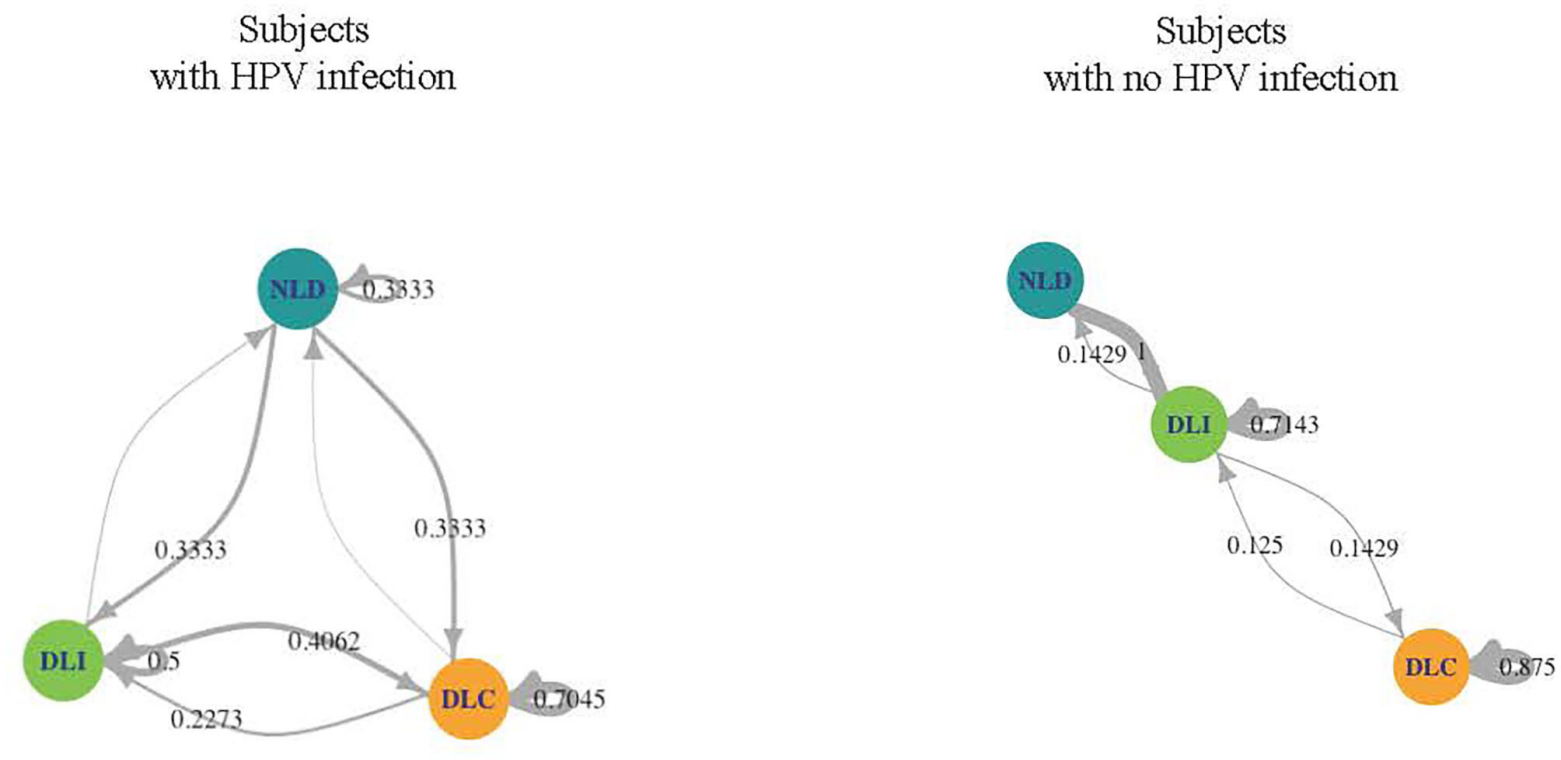
Dynamics of the CW microbiome based on Markov chain modeling. Arrow weights are proportional to the maximum-likelihood-estimate of the transition probabilities between microbiota dominated by *L. crispatus* (DLC), *L. iners* (DLI), or non-*Lactobacillus* (NLD). Women who acquired HPV 16 had higher transition probabilities between microbiota states than women with no history of HPV infection.

We investigated the microbiome temporal stability by analyzing transition probabilities between microbiome community states in the subjects using Markov chain modeling. We found differences between WNHPV and those with HPV16. WNHPV had relatively stable microbiota states (Figure 3). If the subjects had a DLC or DLI microbiota, they were likely to remain in the same microbiota state (88 and 71% probability, respectively) and women who had NDL microbiota had a 100% probability of switching to either DLC or DLI. In comparison, women with HPV 16 infection were more likely to transit between the 3 states than WNHPV and had a higher probability of remaining in NLD.

**Figure 4:**
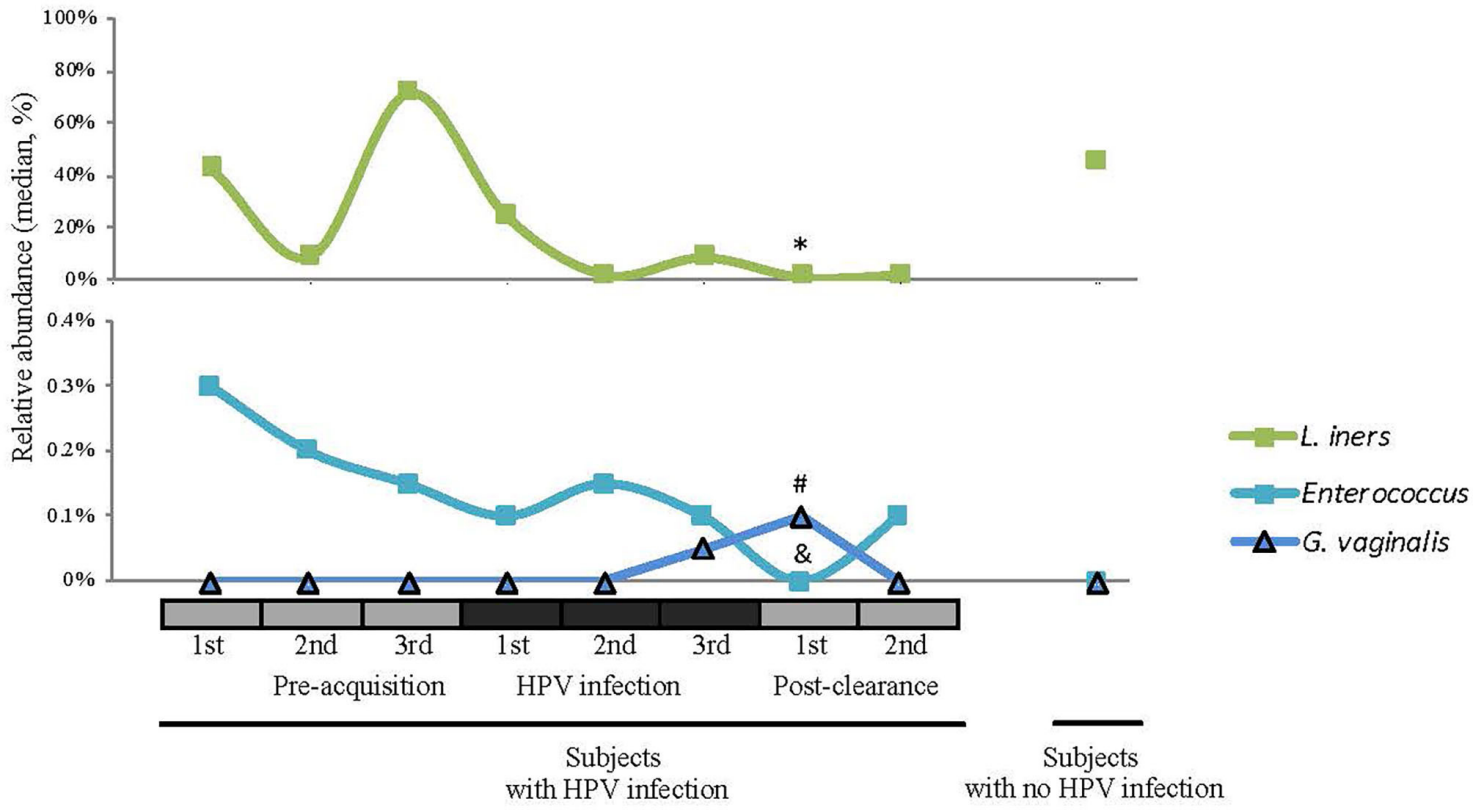
Relative abundances of selected species (*L. iners, Enterococcus, and G. vaginalis)* across different HPV 16 states. Wilcoxon rank-sum test was used in 8 pairwise testing of differences in the bacterial species between immediate post-clearance and the 8 other clinical states including women with no history of HPV. In the immediate post-clearance visit, *L iners* was lower than the 1st (*p = 0.06), 2nd (*p = 0.04), and 3rd (*p = 0.02) pre-acquisition visits, 1st (*p = 0.04), and 3rd (*p = 0.03) infection visits and in women with no history of HPV (*p < 0.001); *G. vaginalis* was higher than in the 1st infection visit (^#^*p* = 0.02) and women with no history of HPV (^#^*p* < 0.001); Enterococcus was lower in all pre-acquisition and infection visits as well as 2nd post-clearance visit (all ^&^*p* < 0.001) but no difference was seen in women with no history of HPV.

We next examined the CW microbiome changes at the species level (Figure 4). Several observations were made for women with HPV16. For *L. iners*, the relative abundance increased at the immediate pre-infection and then decreased thereafter. *G. vaginalis* increased in relative abundance at immediate pre-clearance and significantly increased at post-clearance, which then returned to a baseline months later. Enterococcus had an inverse relationship with *G. vaginalis*. On the other hand, in WNHPV, the relative abundance of *L. iners* was higher than in samples from the immediate post-clearance visits of HPV infected subjects. *G. vaginalis* in WNHPV was lower than immediate post-clearance but similar to pre- and post-infection levels.

### Cytokine Profiles, HPV Status, and Bacterial Species

**Figure 5:**
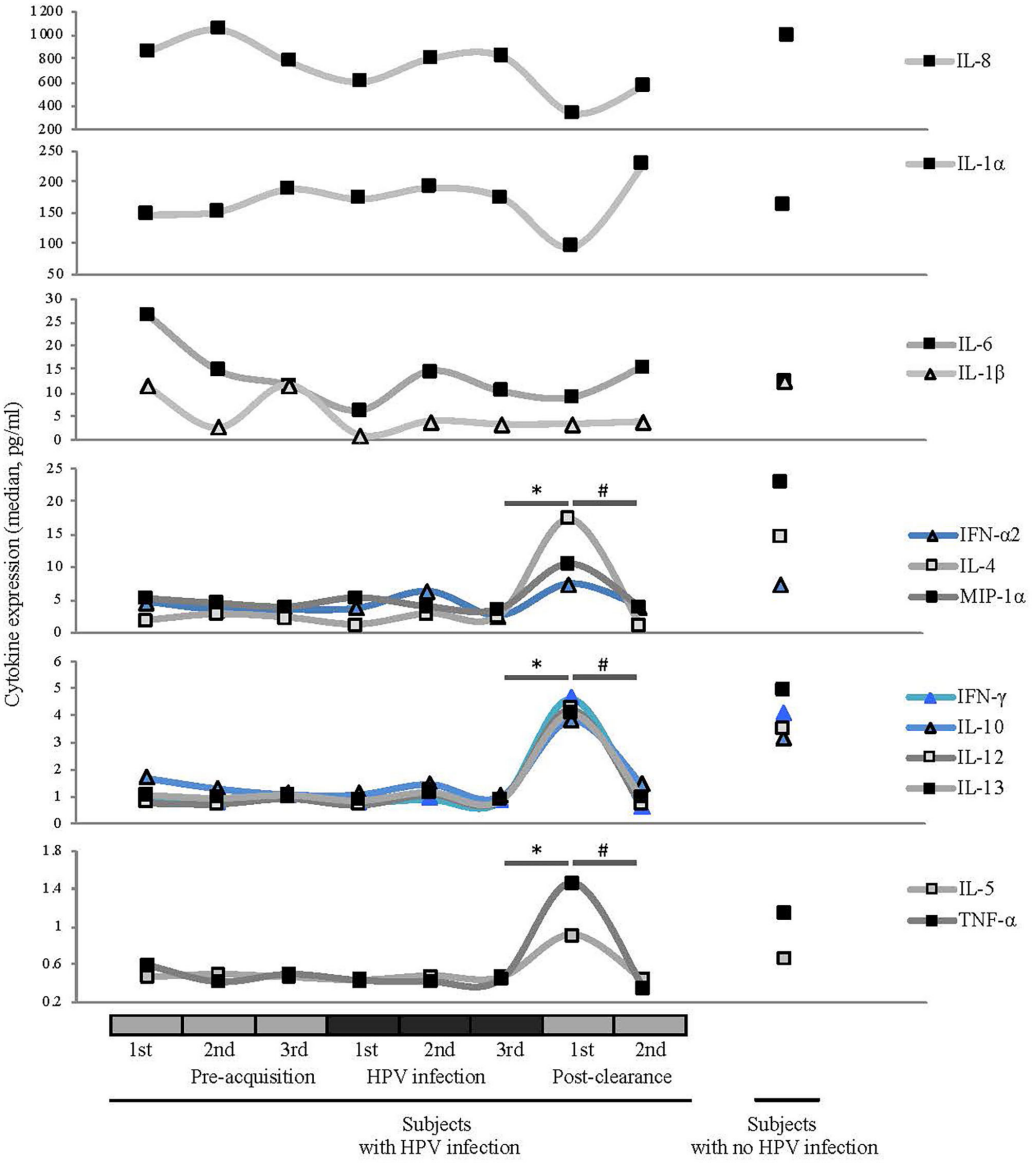
Comparisons of median cytokine expression level (pg/ml) across different HPV 16 states (see Figure 1 for definitions). Wilcoxon rank-sum test was used in 8 pairwise testing of differences in the cytokines between immediate post-clearance and the 8 other clinical states including women with no history of HPV. Cytokines are grouped by the magnitudes of the expression levels. Expression levels of ILs-4, -5, -10, -12, and -13, IFN-γ, IFN-α2, MIP-1α, and TNF-α were significantly elevated at the immediate post-clearance visit compared to all the pre-acquisition and infection visits (all *p < 0.01) and 2nd post-clearance visit (^#^*p* < 0.001). When compared to the immediate post-clearance visit, women with no history of HPV had similar levels of IFN-γ, IFN-α2, and TNF-α, lower levels of ILs-4 (*p* < 0.001), IL-5 (*p* < 0.001), IL-10 (*p* = 0.02), and IL-12 (*p* = 0.02) and higher levels of IL-13 (*p* = 0.02) and MIP-1α (*p* < 0.001).

To investigate the changes in host immune response relative to the HPV status and microbiome changes, we analyzed the expression profiles of 13 inflammatory cytokines listed in Figure 5. Since HPV has been shown to induce both innate and adaptive immune responses, we were interested in examining cytokines essential in both arms. IFN alpha is a key cytokine expressed downstream from innate immune activation. Those associated with an adaptive immune system include interleukin (IL)-12 as its produced from macrophages and dendritic cells, interferon (IFN) α2 produced by natural killer and activated T cells and proinflammatory cytokines such as IL- 6, 8 macrophage inflammatory protein (MIP)−1α, and tumor necrosis factor (TNF) (Stanley, 2010; Daud et al., 2011). Specifically we tested for mucosal expression of candidate antiviral (IFN-α2), type-1 (IFN-γ and IL-12), regulatory (IL-4, IL-5, IL-10, and IL13), and proinflammatory (IL-1α, IL-1β, IL-6, IL-8, MIP-1α, and TNF) cytokines (Scott et al., 2013).

At the immediate post-clearance visit, overall cytokine levels were highly elevated compared to the pre-acquisition and infection visits. The mean number of cytokines ranked in the top quartile based on the expression level was 8.6, compared to the 1.9 to 2.9 in the other visits including the 2nd post-clearance visit months later (all *p* ≤ 0.005; Supplementary Figure 4). When we examined the cytokines individually (Figure 5), we found that the expression levels of IL-4, -5, -10, -12, and -13, IFNγ, IFN-α2, MIP-1α, and TNF-α were significantly elevated at the immediate post-clearance visit compared to pre-acquisition and 2nd post-clearance visit (all *p* < 0.01).

When compared to the samples from the WNHPV, the expression levels of ILs-4, -5, -10, -12, and -13, IFN-γ, IFN-α2, MIP-1α and TNF-α were significantly elevated compared to samples from all the pre-acquisition and infection visits (all *p* < 0.001). When compared to the immediate post-clearance visit, WNHPV had similar levels of IFN-γ, IFN-α-2, and TNF-α, lower levels of IL-4 (*p* < 0.001), IL-5 (*p* < 0.001), IL-10 (*p* = 0.02), and IL-12 (*p* = 0.02) and higher levels of IL-13 (*p* = 0.02) and MIP-1α (*p* < 0.001). The median values are shown in Supplementary Table 3.

**Figure 6:**
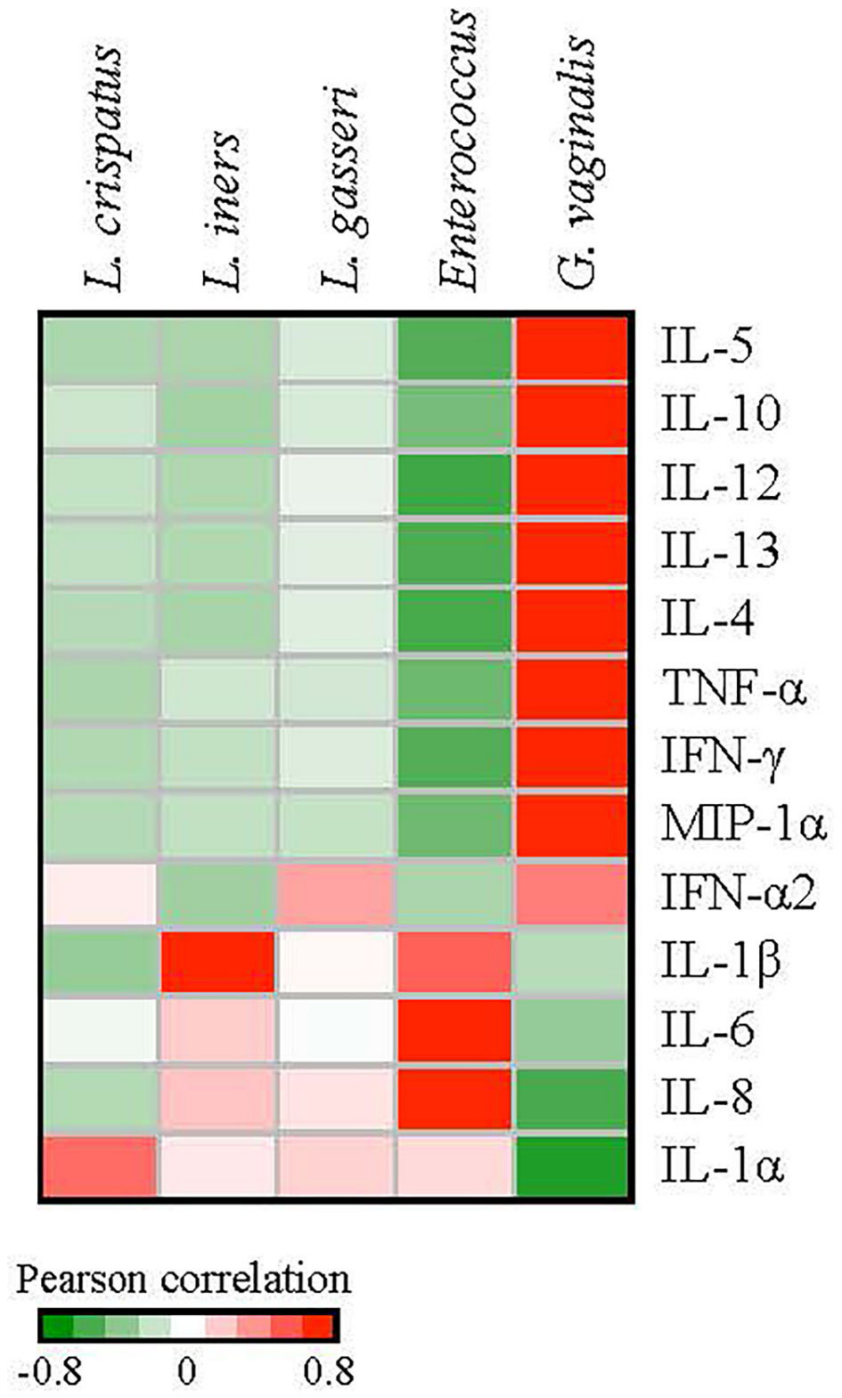
Correlations between the relative abundances of vaginal bacterial species and cytokine expression levels. We used the median values of the relative abundances of bacterial species and the cytokine expression levels among the samples at each visit in the calculation of the Pearson correlation coefficients over the 8 clinic visits. *G. vaginalis* was significantly associated with IL-4 (*p* = 0.003), IL-5 (*p* = 0.004), IL-10 (*p* = 0.02), -IL-12 (*p* = 0.004), IL-13 (*p* = 0.007), TNF-α (*p* = 0.006), IFN-γ (*p* = 0.005), and MIP1-α (*p* = 0.02). *Enterococcus* was associated with IL-6 (*p* = 0.01) and IL-8 (*p* = 0.02). *L. iners* was associated with IL-1β (*p* = 0.02).

We next investigated correlations between cytokines and bacterial species. Pearson correlation coefficients between bacterial species and cytokine expression over eight clinic visits were calculated for the patients with a history of HPV infection. We used the median values of the relative abundances of bacterial species and the cytokine expression levels among the samples at each visit in the calculation. *G. vaginalis* emerged as a bacterial factor associated with several of the elevated cytokines observed in the post-clearance visit including IL-4 (*p* = 0.003), IL-5 (*p* = 0.004), IL-10 (*p* = 0.02), IL-12 (*p* = 0.004), IL-13 (*p* = 007), TNF-α (*p* = 0.006), IFN-γ (*p* = 0.005), and MIP-1α (*p* = 0.02) (Figure 6). Of note, the increase in *G. vaginalis* preceded final clearance and peaked at the time of the observed cytokine peak (Figure 3).

## Discussion

As HPV 16 persistence is associated with the development of several anogenital cancers, it is important to understand mechanisms involved in the clearance of HPV. To our knowledge, ours is the first longitudinal study to examine associations among the cervical-vaginal microbiome, and local immune milieu at HPV 16 acquisition, persistence and clearance. Several findings were worthy of discussion. First, the striking increase in inflammatory cytokine expression was not witnessed until the immediate post-clearance visit. We had hypothesized that we would observe a time period when the response was mounting prior to clearance. Our findings imply that the several-fold increase in the majority of inflammatory markers observed after a documented clearance event was residual evidence of a successful anti-viral response. Since we only obtained samples every 4–6 months, this finding was not surprising in retrospect but emphasizes that the successful immune response is likely to occur suddenly and swiftly and these responses persist for a relatively short period as the expression of the inflammatory markers returned to the baseline months later. Of interest, WNHPV had inflammatory expression levels similar to the post-clearance visit in women with HPV infection. This may suggest that the subjects who acquired HPV 16 were more vulnerable to HPV because of an inadequate immune milieu or response. Another possible explanation is that WNHPV had a previous infection that cleared and now have a higher immune surveillance protecting them from HPV reinfection.

To carry this notion of immune surveillance further, the Markov-chain model of the CW microbiome dynamics analyzed in our study supports the theory that women who acquire HPV 16 have a different local environment than those who appear protected from acquisition. Women who acquired HPV 16 had relatively unstable CW microbiome states in that they had high probabilities of switching from one state to another. In contrast, WNHPV had stable states throughout the visits, predominantly being in DLC and DLI, both of which are considered healthier states than NLD microbiota. The stability observed in the microbiome of this group and yet a higher inflammatory state similar to that observed in the post-clearance state suggest that there is a protective microbiome homeostasis against pathogens.

Second, contrary to our initial hypothesis, the microbiome was not altered dramatically during HPV persistence from the pre-acquisition state. Rather, there were more subtle changes during persistence with initial increases in DLC and emergence of NLD. This shift to NLD became more apparent in the immediate post-clearance shift. As *G. vaginalis* is often more abundant in NLD, the observed increase in *G. vaginalis* during persistence and post-clearance was not surprising. NLD microbiome of which *G. vaginalis* is one of the predominant species, is often considered a pathogenic microenvironment since it has been linked to several morbidities such as pelvic inflammatory disease, premature labor, and HIV acquisition (Ness et al., 2005; Hedges et al., 2006; Libby et al., 2008; Zevin et al., 2016). In the study by Arnold et al. (2016) NLD microbiome was associated with increased inflammation resulting in evidence of epithelial disruption using proteomics. The majority of cytokines found elevated in the post-clearance were also associated with a greater relative abundance of *G. vaginalis*. We hypothesize that HPV infection results in a switch from antimicrobial surveillance, which exists at steady-state, to an anti-viral immune response leading to HPV clearance. This switch may result in loss of microbial control, allowing the expansion of pathogenic bacteria which included *G. vaginalis*. It remains unclear whether NLD microbiome which includes *G. vaginalis* assisted in driving the host immune response resulting in HPV clearance or this was a stand-byer effect. Consistent with our study, Arokiyaraj et al. (2018), reported that *G. vaginalis* was associated with HPV clearance. The recent meta-analysis by Norenhag et al. (2020) found that low Lactobacillus microbiota was more likely associated with HPV and SIL in comparison to the microbiota dominated by *L. crispatus*. These findings are consistent with ours, understanding that most HPV infections and SIL are transient. On the other hand, several other studies found that higher abundances of *L. gasseri, L. iners* and anaerobic species including *G. vaginalis* were associated with HPV infection and HSIL (Gao et al., 2013; Mitra et al., 2015; Shannon et al., 2017). Different from all of these previous studies, which were predominantly cross-sectional, our study investigated women who had persistence but eventually cleared HPV. Our study was not designed to implicate microbiome states or bacterial species that were associated with persistence leading to precancerous and cancer lesions. Therefore, the question remains whether a transient switch in microbiota may be helpful in HPV clearance.

A potential confounder was the fact that 9 women were co-infected with other HPV types in addition to HPV 16. As all women cleared and the samples size was small, we were unable to stratify the analysis. However, HPV co-infections are quite common. We were also unable to examine the role of race/ethnicity which has been shown to influence the microbiome. As we only studied women with HPV clearance, the role of the microbiome in progression remains unknown. None of the women in our studied developed cancer and all women who developed the precancer cervical intraepithelial neoplasia (CIN) 2, 3 were exited for treatment.

In conclusion, this *in vivo* study is the first to document that the clearance of HPV 16 is associated with a distinct inflammatory eruption witnessed immediately after clearance. The inflammatory burst was associated with microbial dysbiosis and an increase in NLD. It is plausible that HPV 16 clearance was associated with anti-viral immune response resulting in a microbiome shift that promoted the dominance of these bacteria in the community. The high stability of the microbiome state observed in WNHPV was associated with a higher level of cytokines as seen in post-clearance state, suggesting that the local microbiome plays a significant role in immune surveillance to maintain an HPV negative state. The use of probiotics to achieve vaginal microbiome stability may be a promising approach to promoting HPV clearance in women with persistent infections.

## Data Availability Statement

The microbiome datasets presented in this study can be found in online repositories. The names of the repository/repositories and accession number(s) can be found at: https://www.ncbi.nlm.nih.gov/, PRJNA588856. Cytokine dataset is available on request.

## Ethics Statement

The primary study was approved by the Committee on Human Research, University of California, San Francisco and patients consented to participate and to place their samples in repository. This study used samples from the repository and was approved by the Institutional Review Board of the University of California, Los Angeles.

## Author Contributions

A-BM was the initial principal investigator of the prospective cohort study and designed this sub-study and oversaw the statistical analysis and prepared the manuscript. HL and BS collaborated in the design of the sub-study and contributed to the manuscript preparation. HL oversaw all the microbiome analysis and all the statistical analyses of the microbiome, cytokine, and metabolome data. BS performed all the microbiome, cytokine, and metabolome data analysis and statistical analyses. HH and EB were essential to the development of the assays for processing the biorepository samples. All authors read and approved the final manuscript.

## Conflict of Interest

The authors declare that the research was conducted in the absence of any commercial or financial relationships that could be construed as a potential conflict of interest.
