# Supplementary material for: Cervical-Vaginal Microbiome and Associated Cytokine Profiles in a Prospective Study of HPV 16 Acquisition, Persistence, and Clearance
Source: Front Cell Infect Microbiol. 2020 Sep 25;10:569022. doi: 10.3389/fcimb.2020.569022 (PMC7546785; doi:10.3389/fcimb.2020.569022)
Supplement: Supplementary file 1 [file Table_1.DOCX]

Supplementary Material

# Supplementary Figures and Tables

## Supplementary Tables

Supplemental Table 1: Number of visits, observation period and race/ethnicity for 14 women with HPV 16 and 8 controls

|  |  | **No. of visits** | **Duration (years)** | **Race** |
| --- | --- | --- | --- | --- |
| Subjects with HPV 16 infection | Subject 1 | 7 | 3 | White |
|  | Subject 2 | 7 | 5 | White |
|  | Subject 3 | 7 | 6 | White |
|  | Subject 4 | 7 | 4 | White |
|  | Subject 5 | 8 | 7 | Latino |
|  | Subject 6 | 7 | 3 | Latino |
|  | Subject 7 | 7 | 7 | Latino |
|  | Subject 8 | 6 | 3 | White Latino |
|  | Subject 9 | 8 | 4 | Latino |
|  | Subject 10 | 7 | 2 | Black |
|  | Subject 11 | 7 | 4 | Asian |
|  | Subject 12 | 8 | 8 | White Latino |
|  | Subject 13 | 7 | 9 | White |
|  | Subject 14 | 5 | 6 | White |

| Subjects without HPV infection | Subject 15 | 4 | 3 | Asian |
| --- | --- | --- | --- | --- |
|  | Subject 16 | 4 | 8 | White |
|  | Subject 17 | 4 | 4 | Asian |
|  | Subject 18 | 4 | 2 | Asian |
|  | Subject 19 | 4 | 5 | White |
|  | Subject 20 | 4 | 5 | White |
|  | Subject 21 | 4 | 5 | Black |
|  | Subject 22 | 4 | 6 | White |

Supplemental Table 2: Samples with microbiome, and cytokine assays


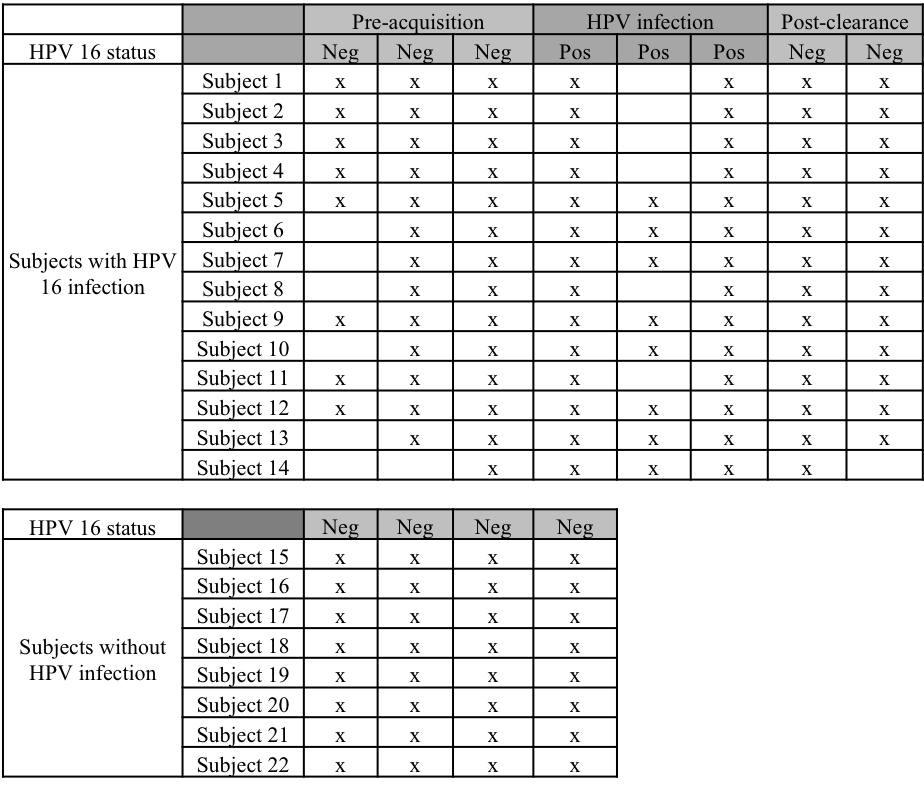


x Samples with microbiome and cytokine assays

Supplemental Table 3: Median Cytokine levels (pg/ml) from pre-acquisition, infection, and post clearance visit samples and in women with no history of HPV infection*

| Cytokine | IFN-α2 | IFN-γ | IL-10 | IL-12 | IL-13 | IL-1α | IL-1b | IL-4 | IL-5 | IL-6 | IL-8 | MIP-1α | TNF-α |
| --- | --- | --- | --- | --- | --- | --- | --- | --- | --- | --- | --- | --- | --- |
| 1st pre-  acquisition | 4.79 | 1.01 | 1.68 | 0.75 | 1.05 | 146.47 | 11.36 | 1.97 | 0.47 | 26.80 | 859.68 | 5.26 | 0.60 |
| 2nd pre-  acquisition | 3.54 | 0.78 | 1.29 | 0.72 | 0.94 | 151.91 | 2.59 | 2.89 | 0.50 | 14.84 | 1046.0 | 4.55 | 0.42 |
| 3rd pre-  acquisition | 3.54 | 1.04 | 1.07 | 0.92 | 1.05 | 188.17 | 11.77 | 2.38 | 0.47 | 11.69 | 770.24 | 4.02 | 0.50 |
| 1st infection | 3.82 | 0.79 | 1.07 | 0.68 | 0.85 | 172.28 | 1.10 | 1.34 | 0.44 | 6.26 | 603.33 | 5.40 | 0.44 |
| 2nd infection | 6.36 | 0.90 | 1.45 | 1.07 | 1.16 | 190.51 | 4.02 | 3.00 | 0.49 | 14.67 | 800.49 | 4.01 | 0.42 |
| 3rd infection | 2.68 | 0.82 | 0.99 | 0.84 | 0.85 | 174.50 | 3.35 | 2.67 | 0.47 | 10.40 | 820.30 | 3.68 | 0.45 |
| 1st post-  clearance | 7.52 | 4.62 | 3.82 | 4.22 | 4.06 | 94.40 | 3.43 | 17.36 | 0.92 | 9.07 | 338.09 | 10.57 | 1.47 |
| 2nd post-  clearance | 4.11 | 0.57 | 1.45 | 0.64 | 0.94 | 227.16 | 3.91 | 1.29 | 0.44 | 15.60 | 569.55 | 3.91 | 0.36 |
|  |  |  |  |  |  |  |  |  |  |  |  |  |  |
| Women with  no history of HPV | 7.52 | 4.06 | 3.17 | 3.50 | 4.91 | 161.77 | 12.45 | 14.84 | 0.67 | 12.53 | 989.20 | 23.19 | 1.16 |

*median cytokine levels in women with no history of HPV infection reflect the combined medians from the 4 samples collected.

Supplementary Figures

**Supplemental Figure 1. Proportion of bacterial species at different HPV 16 states in the women with HPV infection (see Figure 1 for definition) and women with no history of HPV infection ever**. The average of four visits is shown for subjects with no history of HPV infection.

Supplemental Figure 1


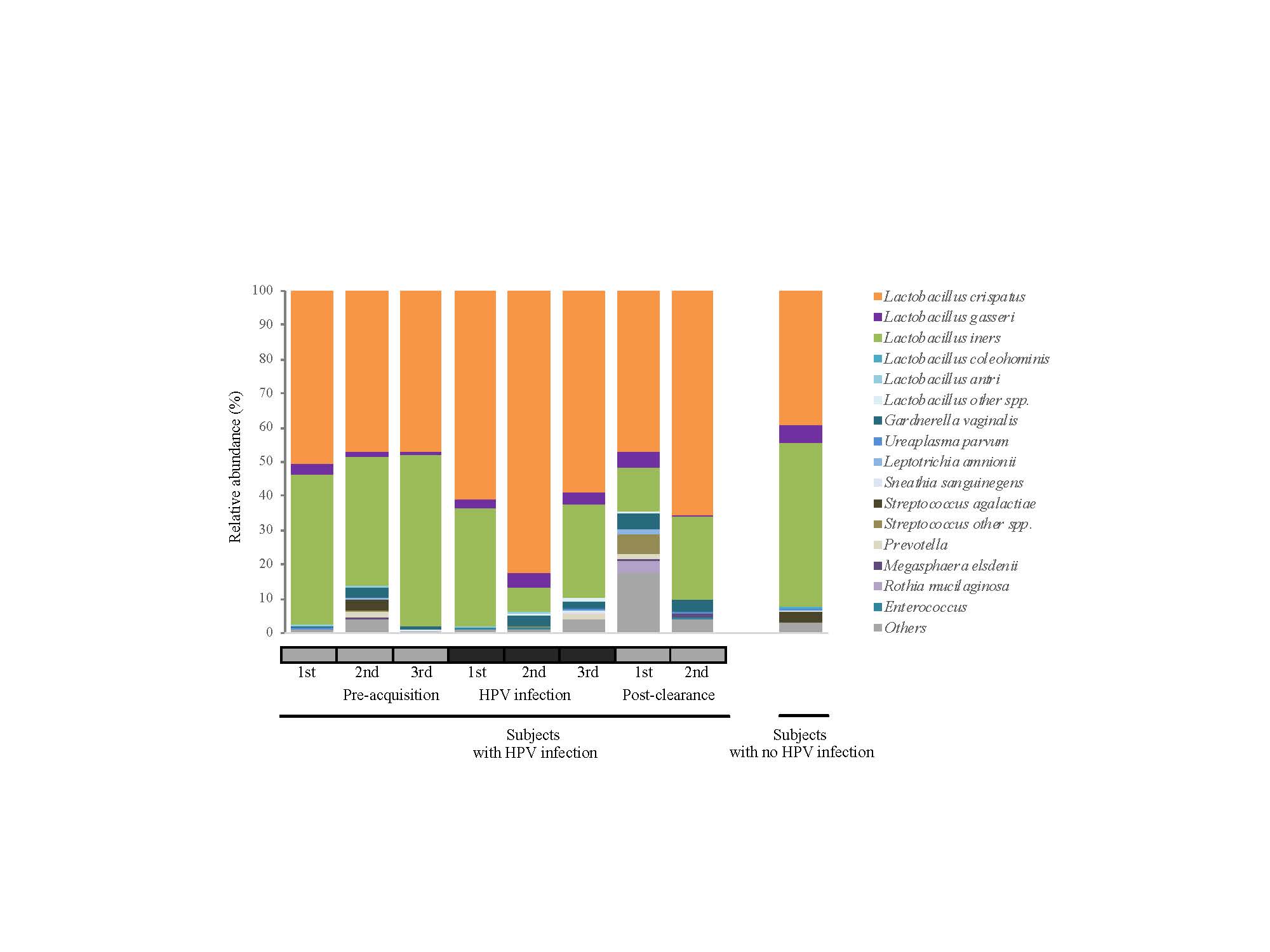


Supplemental Figure 2. **Microbiome composition of the samples ordered** by the relative abundances of the dominant bacterial species detected in the samples. Three microbiota states were recognized: those dominated by *L. Crispatus* (DLC*)* and by *L. Iners* (DLI) and non-*Lactobacillus* dominated (NLD).

Supplemental Figure 2

Supplemental Figure 3. **Proportion of each microbiome dominated state [dominated by *L. Crispatus* (DLC) and by *L. Iners* (DLI) and non-*Lactobacillus* dominated (NLD)] at different HPV 16 states in the women with HPV infection (see Figure 1 for definition) and women with no history of HPV infection ever**. The average of four visits is shown for subjects with no history of HPV infection. More samples from the immediate post-clearance visits had NLD than the pre-acquisition visits combined (Fisher exact; *p = 0.003). This was also true in comparison to samples from women with no history of HPV (Fisher exact test; ^#^p = 0.02).

Supplemental Figure 3

Supplemental Figure 4. **Number of cytokines ranked in the top quartile based on the expression level in each subject at different HPV 16 states (defined in Figure 1)**. Wilcoxon rank-sum test was used in 8 pairwise testing of differences in the numbers of cytokines in the top quartile between immediate post-clearance and the 7 other clinical states. The average number of cytokines from the subjects observed at the immediate post-clearance visits was significantly higher than the average number at each of the other pre-acquisition and infection visits and 2^nd^ post clearance visit (all p values ≤0.005).

Supplemental Figure 4
